# Lipoprotein-induced cell growth and hemocyanin biosynthesis in rhogocytes

**DOI:** 10.1007/s00441-022-03577-1

**Published:** 2022-01-28

**Authors:** Fareed Sairi, Vincent G. Gomes, Fariba Dehghani, Peter Valtchev

**Affiliations:** 1 School of Chemical and Bio Molecular Engineering, University of Sydney, Sydney, 2006 Australia; 2 Department of Biological Sciences and Biotechnology, Faculty of Science and Technology, Universiti Kebangsaan Malaysia, 43600 Bangi, Malaysia

**Keywords:** Rhogocyte cell, Mollusc, Hemocyanin, Flow counting, Flow cytometry

## Abstract

Rhogocyte is a unique molluscan cell that synthesises a supramolecular respiratory protein known as hemocyanin. Its ability to synthesise the protein has eluded the scientists despite hemocyanin’s importance as a carrier protein and complex molecule with anti-viral activity. Although a hypothetical model of hemocyanin release from the rhogocytes lacunae was proposed based on colloid-osmotic pressure mechanism, lack of in vitro studies limits further validation of this model. In this study, we aim to investigate the impact of cell culture conditions and nature of hemocyanin biosynthesis of rhogocyte cells dissociated from *Haliotis laevigata* mantle tissue. Population of cells with different hemocyanin expression levels was profiled using flow cytometry, while hemocyanin concentrations in the media were elucidated by ELISA assay. We demonstrated that addition of lipoprotein supplement into the media resulted in a burst secretion of hemocyanin into the culture media. Over 7 days of culture, the population of cells tagged with hemocyanin antibody increased steadily while hemocyanin release in the media decreased significantly. Variation of culture medium, temperature, growth supplement type and concentration also impacted the cell growth and hemocyanin biosynthesis. These results indicated the possibility of an active process triggered by the addition of supplement to synthesise the protein at the highest amount during the first hour. The current study provides a glimpse of the hemocyanin biosynthesis by rhogocyte that may be significant to understand the cell ability to synthesise supramolecular protein and secretion through lacunae.

**Supplementary Information:**

The online version contains supplementary material available at 10.1007/s00441-022-03577-1.

## Introduction

Hemocyanin biosynthesis occurs in the rhogocyte cells in all molluscan species (Haszprunar 1996; Albrecht et al. 2001; Martin et al. 2011). Apart from hemocyanin biosynthesis, rhogocytes were also suggested to play a significant role in heavy metal haemostasis (Marigómez et al. 2002; Dallinger 2005; Hödl et al. 2010; Kokkinopoulou et al. 2015), nutrient storage (Bani and Delfino 1974) and collagen production (Jones and Bowen 1979). The rhogocyte is an irregular cell embedded in connective tissues and found mostly in the midgut gland and mantle tissue of molluscs (Albrecht et al. 2001). The cell morphology observed in tissue sections is predominantly round and elongated with 10 to 30 µm in size with a layer of basal lamina covering each individual cell. The cell also includes extracellular lacunae space (ELS) through the formation of invaginations under the cell membrane covered by cytoplasmic bars (Boer and Sminia 1976; Haszprunar 1996; Kokkinopoulou et al. 2015). Several studies also reported that hemocyanin-like particles accumulated in the ELS and cell’s cisternae of several gastropods (Albrecht et al. 2001; Dallinger 2005; Martin et al. 2011).

Previously, we were able to detect abundant rhogocyte cells in dissociated *Haliotis laevigata* mantle tissue using flow cytometry targeting localised hemocyanin inside the ELS (Sairi et al. 2015). Two groups of rhogocyte cells with a different profile of type 1 hemocyanin mRNA and immunocytochemistry profiles were observed. The result also suggested that the mantle tissue’s rhogocyte cells only produce one type of hemocyanin and exist in active and resting states. The active state is when the cells readily synthesizes and releases hemocyanin into the ELS, which was reflected by low hemocyanin mRNA transcription in the cytoplasm, but prominent and distributed hemocyanin signal around the cell membrane detected by immunocytochemistry. The resting rhogocyte state manifests with a high transcription hemocyanin mRNA in the cytoplasm and distinct localised hemocyanin accumulation around the cell membrane. Our findings corroborated those of Kokkinopoulou et al. (2015) who proposed the colloid-osmotic pressure mechanism as a hypothetical model to explain the hemocyanin release from the rhogocyte cell. The proposed model suggested that the accumulation of hemocyanin in the ELS results in water to diffuse into the ELS until it reaches a certain osmotic threshold. Following that, the built-up pressure induces contraction of the actin-rich peripheries of the cytoplasmic bars and releases the ELS content.

The proposed hypothetical model of colloid osmotic pressure mechanism, however, only addresses the final release of hemocyanin. The factors affecting the biosynthesis of hemocyanin including the gene regulation and post-translation modifications process are still obscure. The lack of knowledge regarding the rhogocyte cell culture and growth characteristics also adds to the difficulty in elucidating hemocyanin biosynthesis. To date, only Kokkinopoulou et al. (2015) and Streit et al. (2005) studied rhogocyte population response to heavy metal-induced stress by *Lymnaea stagnalis* and the expression of type I and II hemocyanin during *Haliotis asinia* trochophore and pre-torsional veliger stage, respectively*.* Streit et al. (2005) suggested that rhogocytes were to be mesenchymal cells, and this has also been corroborated by several other studies (Albrecht et al. 2001; Martin et al. 2011; Kokkinopoulou et al. 2015). Whether these cells could proliferate into similar cell types or other cells differentiate into rhogocytes is still debatable.

Our previous study demonstrated a successful decellularisation of molluscan tissue and distinguished rhogocyte population using immunocytochemistry approach and flow cytometry analysis (Sairi et al. 2015). Therefore, it is now possible to investigate the effect of different media and growth supplements on the growth pattern of rhogocyte cells and specifically the hemocyanin biosynthesis or release in detail never achieved before. Formulation of culture media and effective supplements is still one of the major bottlenecks in developing marine invertebrate cell culture. The difficulty is due to the lack of vital information regarding the cell requirements and their physiology (Rinkevich 2005, 2011). Several studies had attempted to establish a marine invertebrate primary culture with limited success (Lebel et al. 1996; Giard et al. 1998; Sera et al. 2000; Suja et al. 2017). The lack of knowledge on rhogocyte cell physiology has been attributed to the difficulty of isolating and sorting out rhogocyte cells and the poor understanding of optimal culture conditions. Therefore, advancing this field will improve our understanding of rhogocyte cell morphology, physiology, capability to synthesise a supramolecular protein will provide further insights into the animal’s biology and unique immune system. This study aims to elucidate the effect of different growth parameters including media composition, growth supplements and temperature on the growth of rhogocyte cells in vitro. The results of this study could provide additional support to the colloid osmotic pressure model of hemocyanin biosynthesis and assist the development of better media for marine invertebrate cell culture in general.

## Materials and methods

### Materials

Juvenile greenlip abalone (*Haliotis laevigata*) were farmed in Tasmania and distressed for a week without feeding in 4000-l tanks filled with filtered sea water at 14–18 °C. Dispase, sodium chloride (NaCl), potassium chloride (KCl), calcium chloride (CaCl_2_), CelLytic M reagent, ethidium homodimer, phenazine methosulfate (PMS), 3,3′,5,5′ tetramethylbenzidine (TMB), bovine serum albumin (BSA) and ammonium sulphate were purchased from Sigma-Aldrich, Australia. Phosphate buffer solution (PBS pH 7.4), antibiotic–antimycotic solution (anti-anti) 100 × , fetal bovine serum, KnockOut™ serum replacement, minimum essential medium (MEM) vitamin solution 100 × , minimum essential medium (MEM) amino acid solution 100 × , chemically defined lipid concentrate, (2,3-Bis-(2-Methoxy-4-Nitro-5-Sulfophenyl)-2H-Tetrazolium-5-Carboxanilide (XTT) assay kit, Insulin-Transferrin-Selenium-Ethanolamine (ITS -X) (100 ×), fluorescein isothiocyanate (FITC)-conjugated anti-rabbit antibody from goat and Qubit 2.0 Fluorometric Protein Quantification kit were supplied by Invitrogen (US). F-12 nutrient mixture ham medium, Leibovitz’s L-15, modified Eagle’s medium (MEM) and Dulbecco’s modified Eagle’s medium (DMEM) were supplied by Gibco, Lipimax® by Selbourne Biological Service (Aus), magnesium chloride and magnesium phosphate by MERCK. Rabbit anti-hemocyanin polyclonal antibody was produced in-house.

### Confocal imaging

Cells were observed using Olympus FV1000 at 60 × magnification with UPLSAPO 60 × W; NA: 1.20 objective lens. Fluorescence signal acquisition was conducted using blue 405-nm laser to excite DAPI and emission filtered with dichroic mirror SDM490 nm and blue 488-nm laser to excite FITC signal with emission filtered by dichroic mirror SDM650 nm, laser power for 405 nm, 488-nm laser, 633-nm laser was 1% with 400–500 PMT voltage value, 3% with 400–500 PMT voltage value and 10–12% with 500–600 PMT voltage value, respectively. Images were taken with 12 bits/pixel and viewed using the Olympus Fluoview version 1.7a viewer software.

### Enzymatic dissociation of mantle tissue

Single-cell suspension of mantle tissue was prepared with enzymatic dissociation using 2.4 U/ml dispase as described in Sairi et al. (2015). Briefly, the abalone was bled out by inflicting a small incision at the foot. The animal was then placed in a sterile plastic bag and left to bleed out on ice for an hour. After bleeding out the hemolymph, the abalones were rinsed with sterile distilled water to remove dirt and slime before mantle strips were excised with a scalpel. The mantle strips were rinsed with distilled water a few times, immersed in 70% alcohol for 1 min and rinsed again with sterile distilled water. The sterilisation was repeated twice before the mantle strip was washed with phosphate buffered saline (PBS pH 7.4). Finally, the mantle strip was minced finely with scissors. Minced tissue was subjected to 2.4 U/ml dispase and digested for 2 h at 25 °C with gentle shaking. Dissociated cells were then strained with 40-µm cell strainers and centrifuged at 190 g for 10 min. The resulting cell pellet was washed and subjected to flow cytometric counting.

### Immunofluorescence and live/dead assay staining

Prior to flow cytometric counting, dissociated and cultured cells were stained with anti-hemocyanin antibody and a live/dead assay reagent, ethidium homodimer (EtH). Briefly, 0.5 ml of cell suspension was collected from each tube or flask and mixed with 1 µl of EtH, followed by 30 min incubation at 25 °C. After 30 min incubation, the cell suspension was centrifuged at 190 g for 10 min. In order to count the rhogocyte cell only, the cells were stained with anti-hemocyanin antibody and FITC-conjugated secondary antibody. The cell pellet was first resuspended in PBS at 1:1000 dilution of anti-hemocyanin antibody and incubated for 1 h. The suspension was then centrifuged, and the cell pellet was washed with PBS prior to resuspension with 1:1000 FITC-conjugated anti-rabbit antibody and then again incubated for 1 h. Finally, the cell suspension was centrifuged, resuspended in 0.5-ml PBS and subjected to flow cytometry analysis.

### Flow cytometry counting and cell viability

Flow cytometry counting and viability assay were performed using Accuri C6 flow cytometer based on the volumetric method which enabled cell counting by collecting the number of events in a defined volume. Following cell staining with anti-hemocyanin antibody and live-dead assay reagents such as ethidium homodimer (EtH), the suspension was then strained with a 40-µm strainer into a 5-ml FACS tube and subjected to flow counting. A threshold limit of FSC-H channel was set at 11,000, SSC-H channel at 5000, core flow rate (20 µl/min) and a fixed sampling volume of 100 µl. The flow cytometry was set to run with distilled water to gate out resulting noise from debris using FSC-H vs. SSC-H plot. Fluorescence channel was set to FL1-H (488-nm laser/515-nm filter) and FL2-H (515-nm laser/635-nm filter). Initial gate for negative events was set by running unstained cells using FL1-H vs. SSC-H and FL2-H vs. SSC-H, respectively. Subsequently, gates were set to include positive events. The gates were also set to collect cell events excluding aggregates and debris. Cell yield and viability were determined through the FL1-H vs. FL2-H plot.

### Heterogeneous primary cell culture

Cells dissociated from the mantle tissue were used to establish a heterogeneous primary culture of rhogocyte cells. Typically, mantle tissues from three abalones were pooled and dissociated using 2.4-U/ml dispase for 2-h at room temperature (Sairi et al. 2015). After dissociation, cells were strained with a 40-µm strainer to remove large debris and pelleted at 190 × g centrifugation to remove enzyme solution from cells. After centrifugation, the cells were resuspended in PBS and centrifuged again to wash the residual enzyme. In the media selection study, five different basal media including Ham F12, Leibovitz L-15, Medium 199, minimum essential medium (MEM) and Dulbecco minimal Eagle’s medium (DMEM) were used. The seeding density for each media was adjusted to 4 × 10^5^ cells/mL in T25 flasks. The cells were cultured for 9 days at 25 °C. To identify optimal growth supplement, cells were cultured in MEM media with four different primary supplements (10% fetal bovine serum (FBS), 10% Lipimax, and 10% Knockout serum). The seeding density however was adjusted to 5 × 10^4^ in T25 flasks, and cells were cultured for 9 days at 25 °C. Cells were also grown at 17 °C and 25 °C to investigate the impact of temperature. To evaluate hemocyanin biosynthesis in vitro, cells were cultured with growth supplement at 17 °C. All media were supplemented with 1% antibiotic–antimycotic solution (anti-anti) and 1 × concentration of modified salts as shown in supplementary Table S1.

### XTT assay

Cell viability was examined using XTT/PMS assay, described in detail by Roehm et al. (1991) with slight modifications. Briefly, 1 mg/ml of XTT solution was freshly made by dissolving XTT in hot F12 medium (60 °C). A stock solution of 100-mM Phenazine methosulfate (PMS) in PBS was made and stored in 4 °C up to 1 month. A final concentration of 25-µM PMS was mixed with XTT solution prior to assay. A total of 25-µl XTT solution was then added to each 100-µl culture, followed by 6 h of incubation in 25 °C incubator. The absorbance was measured at 450 nm with a reference wavelength of 690 nm. Absorbance from blanks, which consisted of media and XTT/PMS without cells, was deducted from the sample reading.

### Determination of hemocyanin concentration

ELISA was used to determine the amount of hemocyanin detected in media during in vitro cell culture of rhogocyte cells. A standard curve of hemocyanin was prepared by performing two-time serial dilution on solution of 22 mg/ml of hemocyanin. In order to normalise the background reading from the media, hemocyanin was diluted using the same media used for cell culture. Three background controls were set: a well without hemocyanin treated with both antibodies (C1), a well with 22 mg/ml hemocyanin treated with primary antibody only (C2) and a well with 22 mg/ml hemocyanin treated with secondary antibody only (C3). Final OD reading was calculated according to the formula showed in Eq. (1). Readings from C2 and C3 were always lower than C1. Thus, both of them were not included as blanks.

Equation 1: ELISA optical density reading

$${OD}_{450-650}= {RAW OD}_{450-650}-{\mathrm{C}1 OD}_{450-650}$$

Samples were prepared by collecting 5-ml of media from T25 flasks and centrifuging it at 570 × g to separate media and cells. The media was collected into a new tube, while the cell pellet was further treated with 500 µl of CelLytic M reagent (Sigma) for 1 h at room temperature or overnight at 4 °C. After incubation, lysed cell pellet was centrifuged at 1000 g for 15 min. The resulting supernatant was transferred to a new tube and used immediately or stored in − 80 °C for long-term storage. For ELISA, 100 µl of solution from the media or the cell supernatant from the lysed cell was allocated into a 96-well plate in triplicates together with the standards. The plate was incubated for 1 h at room temperature or overnight at 4 °C to allow the protein to adsorb to the well plates. After incubation, the plate was subjected to blocking with 0.5% albumin solution prepared in PBS (pH 7.4) for 1 h. After blocking, the wells were incubated with 100 µl of 1:20,000 dilution of rabbit-anti-hemocyanin antibody for another hour. After primary antibody incubation, the wells were washed with PBS twice to remove excess antibody prior to the addition of secondary antibody, 1:20,000 dilution of anti-rabbit IgG conjugated with peroxidase. The standard curve was fitted using non-linear regression fit and sigmoidal dose–response in GraphPad Prism software version 6 (www.graphpad.com).

### Statistical analysis

Data were presented as mean ± SEM, and statistical significance was determined using one-way ANOVA for single comparisons and two-way ANOVA for multiple comparisons, followed by Tukey–Kramer post hoc test. The analysis was performed using GraphPad Prism version 6.00 for Windows (San Diego California USA). Statistical significance was accepted at *P* < 0.05 and indicated in the Figures as * (*P* < 0.05), ** (*P* < 0.01), *** (*P* < 0.001) and **** (*P* < 0.0001).

## Results

### Microscopic observation of heterogeneous cell culture

**Fig. 1:**
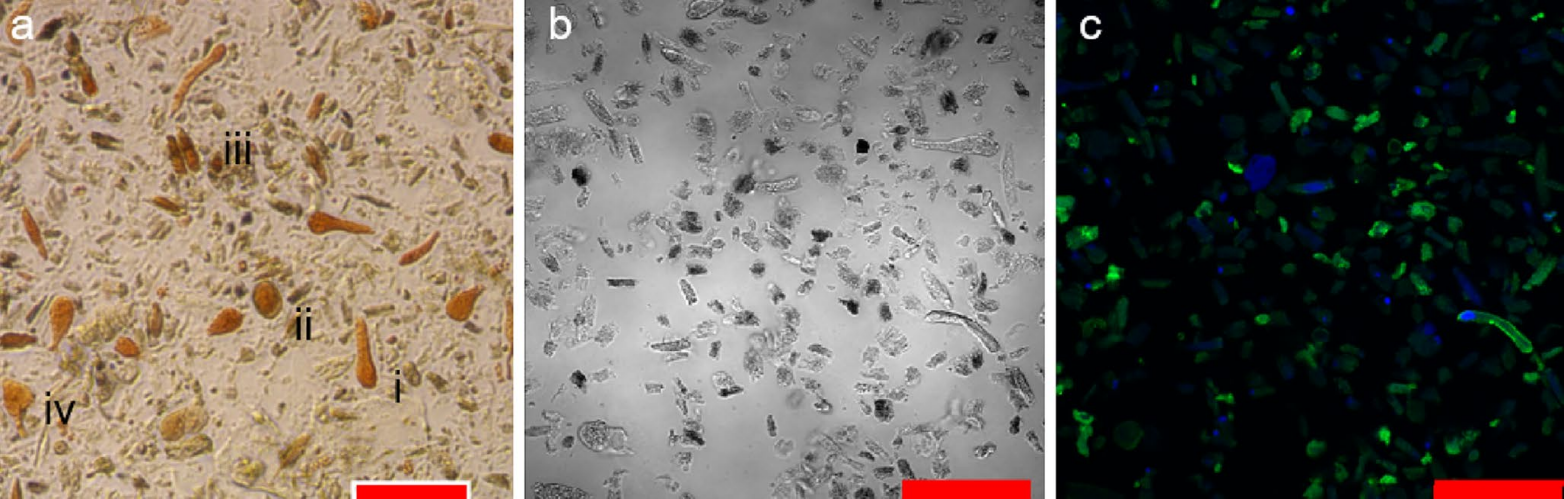
Light microscope images of metabolically active cells (orange) cultured in MEM after XTT assay (**a**). Cell types include (i) elongated, (ii) round, (iii) ovoid and (iv) irregular. Phase contrast image of dissociated cells (**b**) stained by anti-hemocyanin antibody/FITC and DAPI showing rhogocyte cells in green (**c**). Red scale bar = 50 µm

Microscopic analysis revealed various cell phenotypes including round, ovoid, irregular and elongated, which varied in size (Fig. 1a). Cells observed after final treatment during the XTT assay revealed a various degree of orange colouration due to deposition of reduced formazan, which indicates significant differences in metabolic activity. Morphological characterisation was not adequate to identify the rhogocyte cells unambiguously. Therefore, cells were tagged with anti-hemocyanin antibody and observed using a confocal microscope. Rhogocyte cells were detected by fluorescence microscopy as bright green fluorescence spots in Fig. 1b, c. However, their lack of morphological similarities complicates the cells characterisation or detection. Therefore, an automated approach using flow cytometry analysis was used to detect these cells.

### Effect of basal media on cell growth

**Fig. 2:**
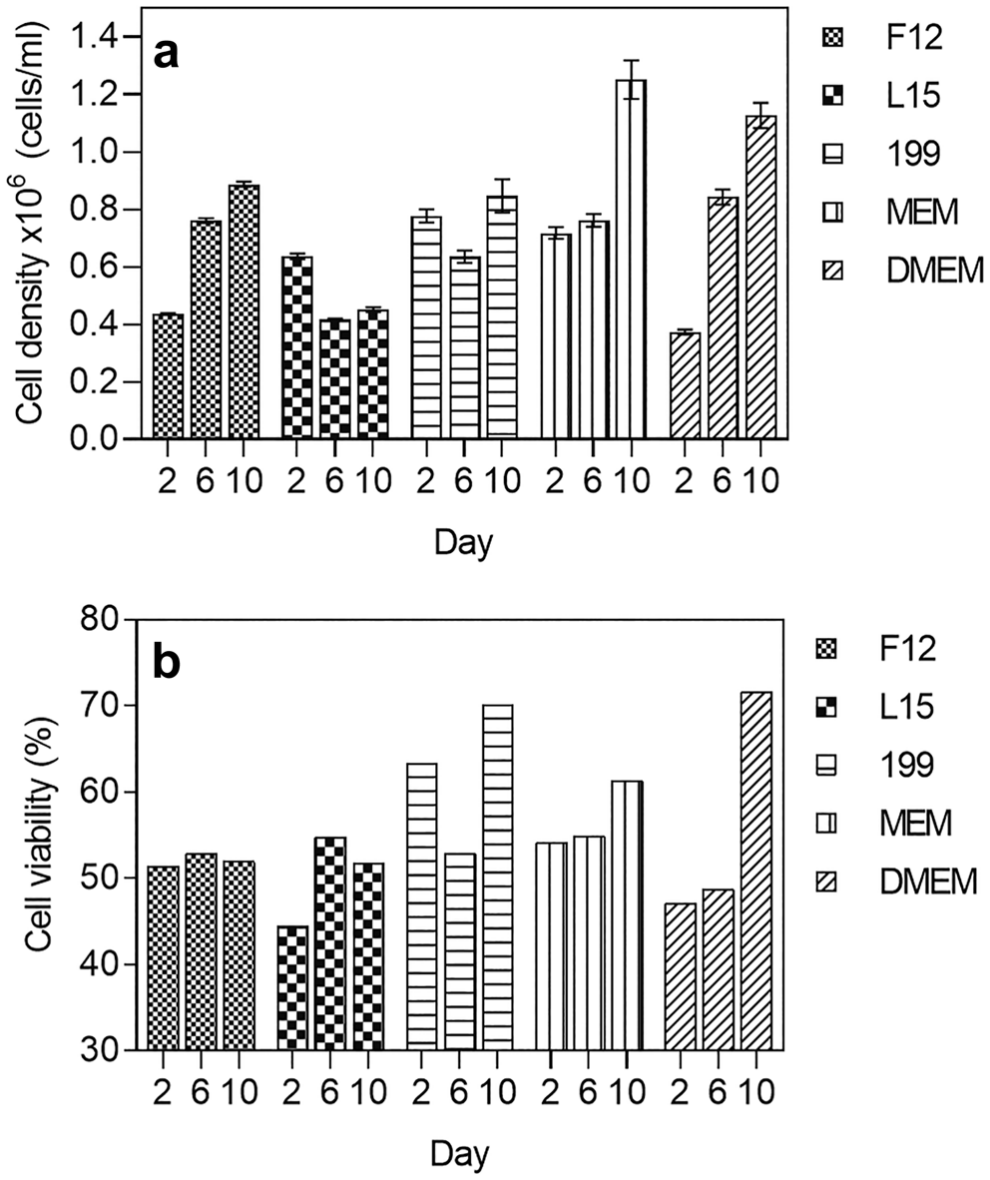
Cell counts by flow cytometry of live rhogocyte cells cultured in different basal media for 10 days. **a** Rhogocyte cell concentration (stained by haemocyanin antibody), **b** cell viability determined by ethidium homodimer

**Fig. 3:**
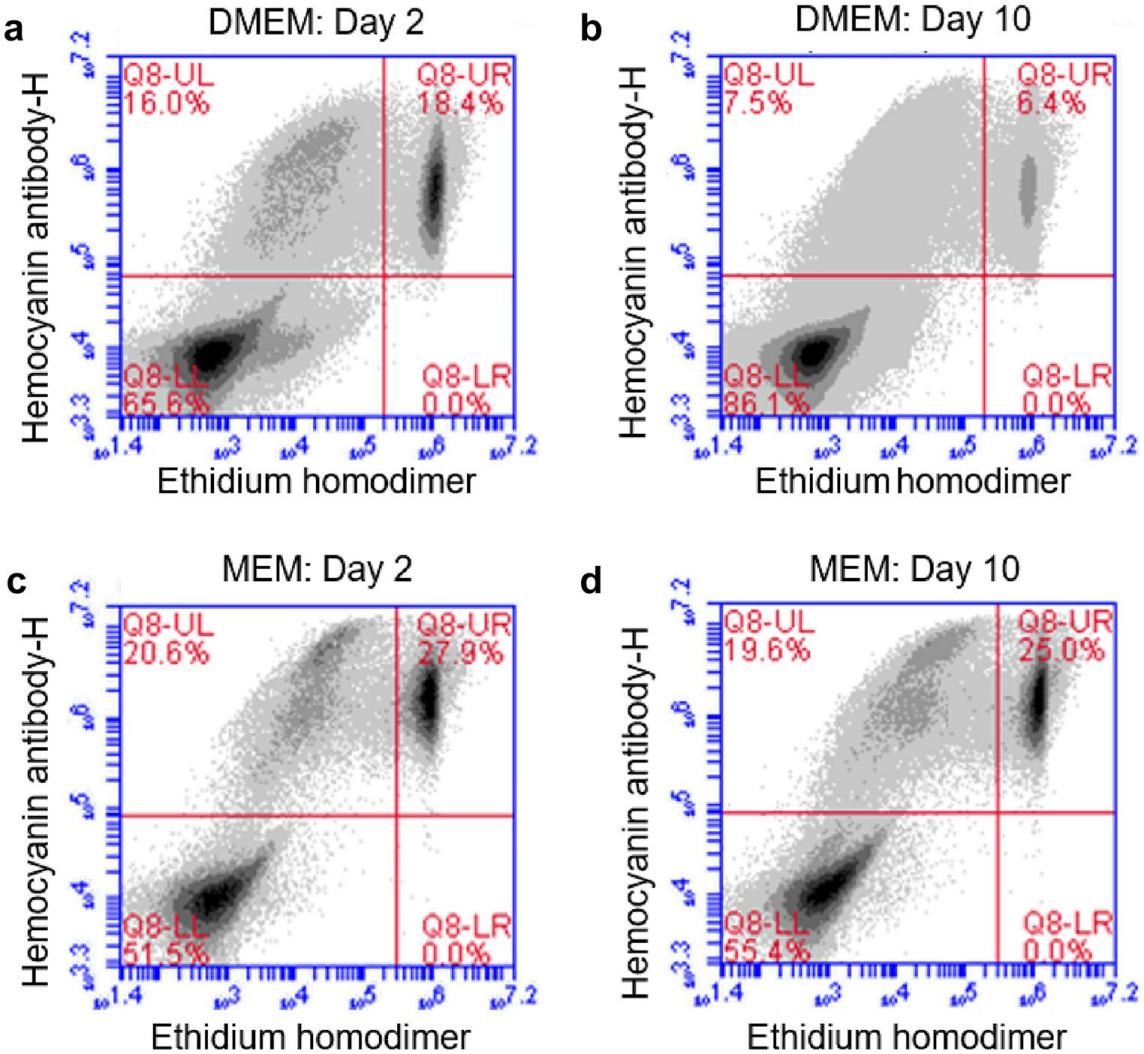
Presence of cell debris in bottom left quadrant when cells were cultured in DMEM and MEM with 10% Lipimax. Cultivation in DMEM on day 2 (**a**) resulting to 65.6% debris and increased to 86.1% on day 10 (**b**). When using MEM media, the cell debris were at 51.5% on day 2 (**c**) and slightly increased to 55.4 on day 10 (**d**)

The effect of different basal media and growth supplements on heterogeneous cell culture were analysed using flow cytometry. A gating strategy was used as depicted in Fig. S1 to select the rhogocyte cell population. The media used in this study included DMEM, MEM L15, F12 and 199. As depicted in Fig. 2a, for the average mean of all time points, in each treatment within flow cytometry analysis MEM resulted in a significantly higher cell yield compared to the rest of the media (*P* < 0.005). At day 10, MEM resulted in 3.1-fold of cells growth compared to the number of seeded cells (Table 1) despite reduced cell viability compared to DMEM (Fig. 2b). In this study, MEM was a better media for rhogocyte cell culture compared to DMEM. As depicted in Fig. 3a, b, the accumulation of the extra-cellular matrix caused a higher ratio of events detected in the flow count as debris (DMEM days 2 and 10, lower left quadrant). After 10 days of culture, the amount of debris detected in the lower left quadrant increased by 20% which lowered the efficiency of detecting cell events in the upper left and right of the quadrant. When MEM were used as media, the lower left quadrant only showed < 5%

**Table 1:** Highest cell fold increase of rhogocyte cells cultured with different basal media

Basal media	Highest cell fold increase	Day of highest fold
Ham F12	2.22	10
Leibovitz L-15	1.59	2
Medium 199	2.12	10
Minimum essential medium (MEM)	3.13	10
Dulbecco minimal Eagle’s medium (DMEM)	2.82	10

### Effect of growth supplements on rhogocyte cell growth

**Fig. 4:**
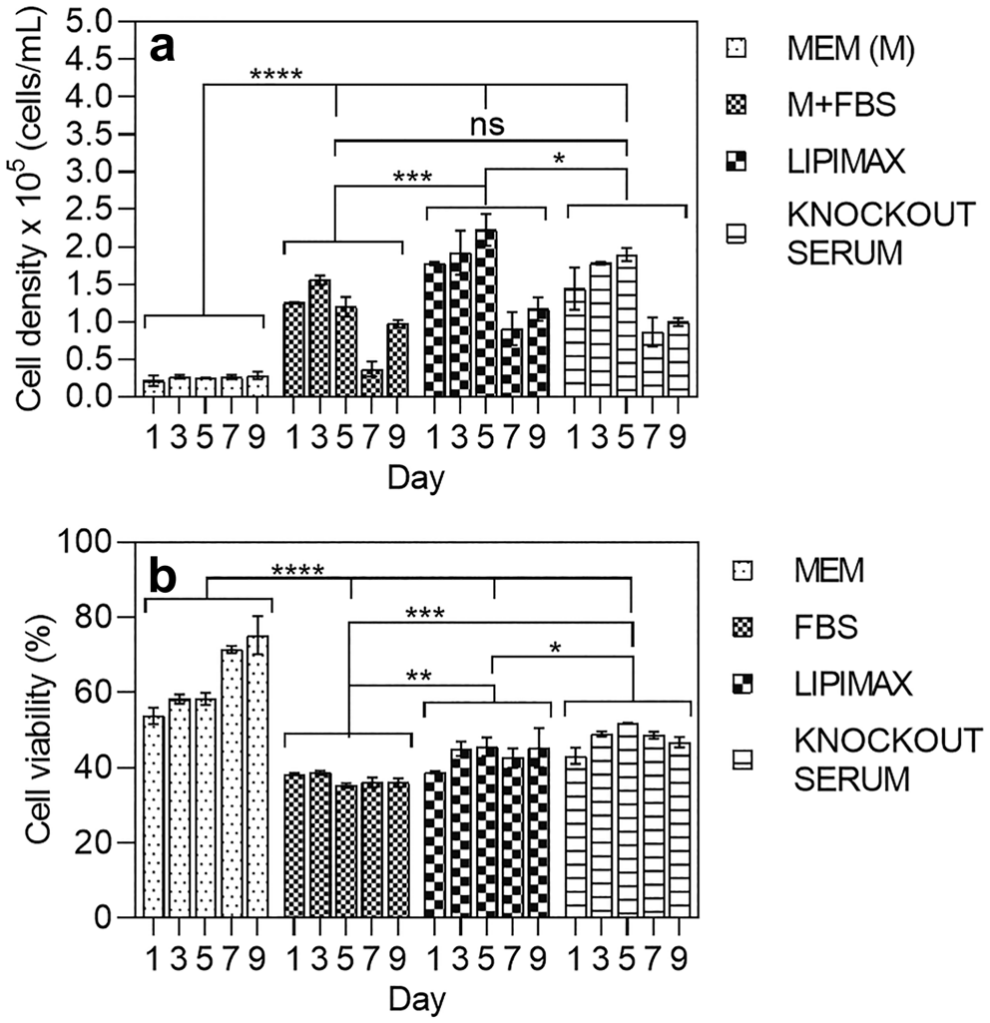
Evaluation of growth supplement at 10% concentration with MEM as basal media. **a** Cell concentration and **b** viability determined by flow cytometry. ****P < 0.0001, ***P < 0.005, **P < 0.05, *P < 0.5, ns, not significant

**Fig. 5:**
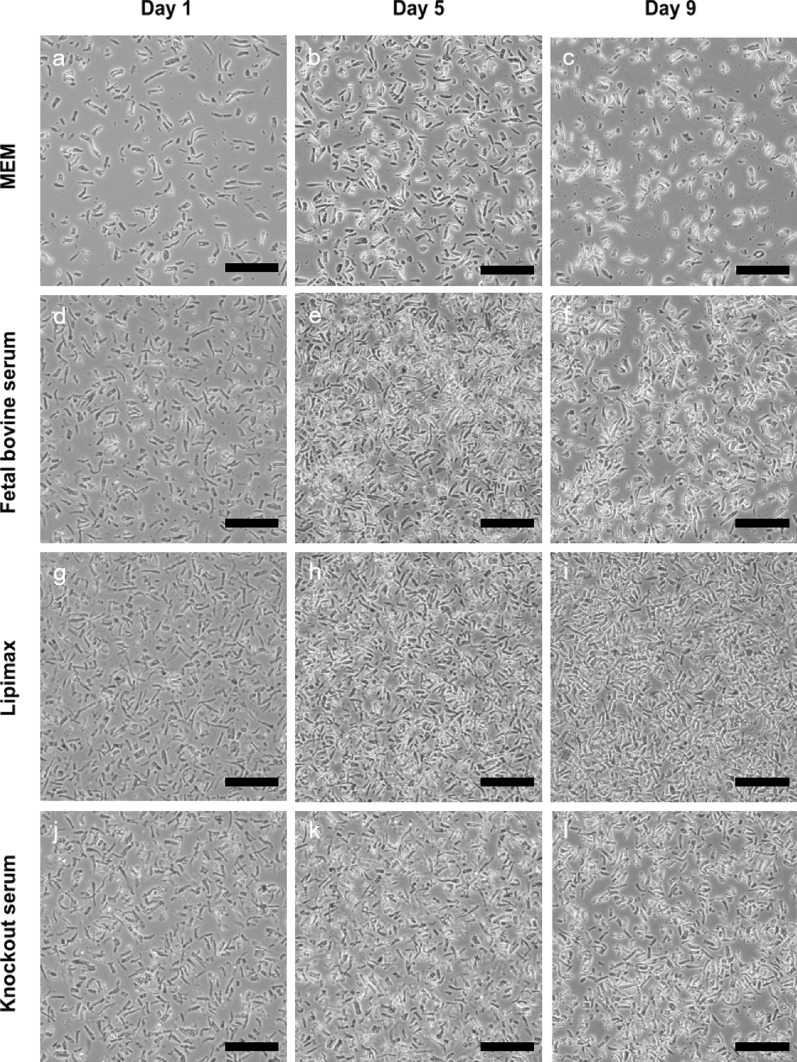
Phase contrast microscope images (200 ×) of cells cultured in different growth supplements for 9 days. Scale: 100 µm

After selecting the optimal base media, the effect of growth supplements on rhogocyte cell growth was investigated. Supplements such as fetal bovine serum (FBS), Lipimax (high lipoprotein extract) and Knockout serum were added to MEM medium at 10% (v/v). As depicted in Fig. 4, the rhogocyte cell growth was significantly higher when Lipimax was added to the MEM compared to FBS and Knockout serum. In 5 days of culture, the cell yield increased by 10, 9 and sevenfold for Lipimax, Knockout serum and FBS, respectively compared to MEM without supplement (Table 2). The cell number increased gradually until day 5 and decreased dramatically at day 7. However, the sudden drop of rhogocyte population was not observed in media without supplements. The cell viability was between 40 and 50% throughout the culture period, except for MEM without supplement (Fig. 4). When media supplement was added, the cells formed a dense population surrounded by extracellular matrix (Fig. 5h, i). Finally, cells were negatively affected by abalone hemolymph that was used as supplement at 1% and 10% v/v (data not shown).

**Table 2:** Highest cell fold increase when growth supplement was included at 10% concentration (v/v)

Supplement (10%)	Highest cell fold increase	Day of highest fold
Control	1.33	9
Fetal bovine serum	7.31	3
Lipimax	10.46	5
Knockout serum	8.90	5

**Fig. 6:**
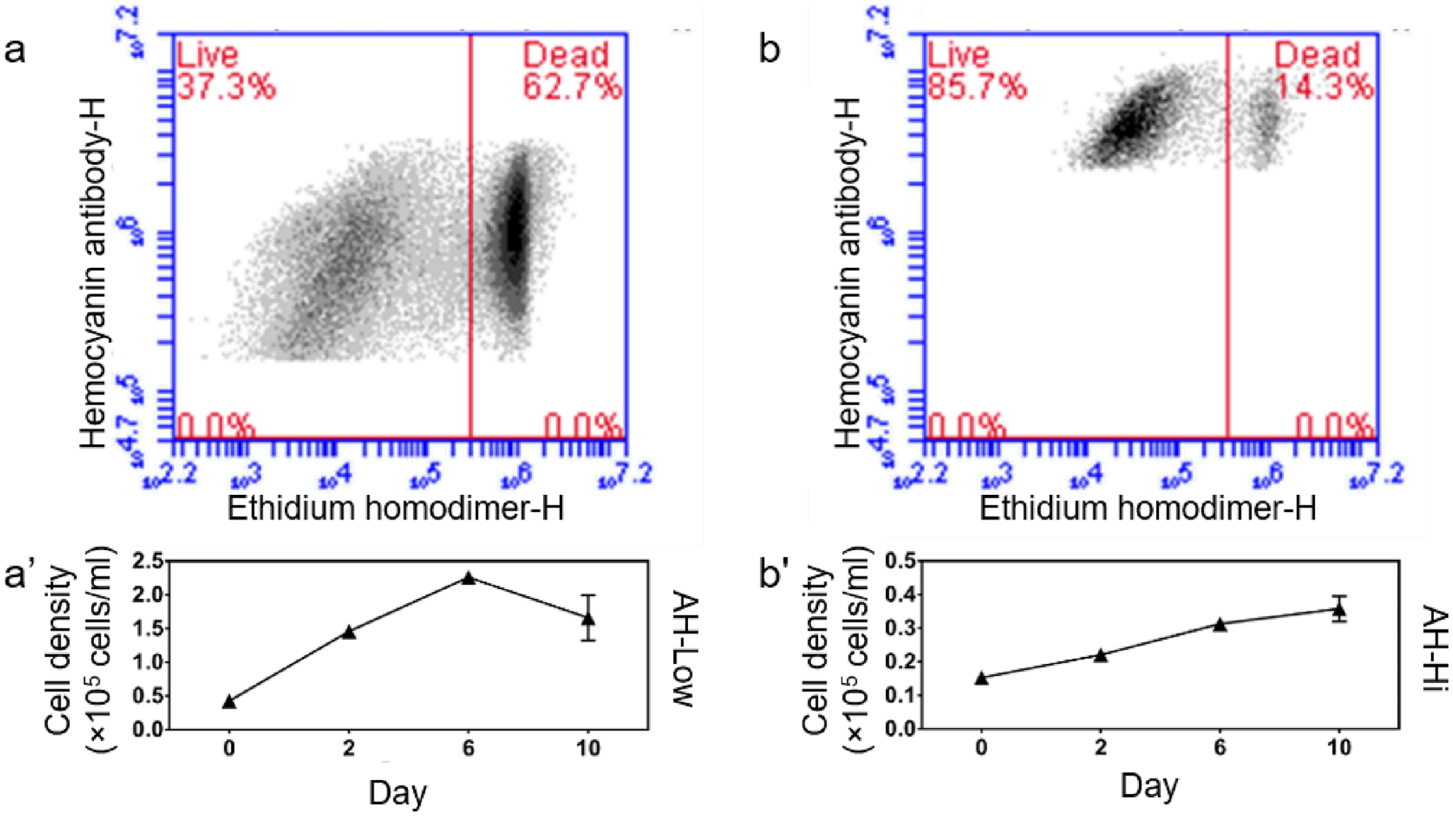
Flow cytometry analysis of cell population with low and high hemocyanin antibody signal. **a** Scatter plot of low hemocyanin signal population and **b** high hemocyanin signal population. **a'** Cell growth of population with low hemocyanin signal and **b’** high hemocyanin signal

The flow cytometry analysis also provided additional perspective of cell population density based on signal profiles (Fig. 6a, b). As depicted in the graph of Fig. 6a′, cells with low hemocyanin (by immunocytochemistry) signal grew steadily up to day 6 before, cell number decreased whilst cells with higher hemocyanin signal continued to grow steadily up to day 10. Although the population of cells with high hemocyanin signal was lower in number, they were more viable (unstained by ethidium homodmer in upper left quadrant) compared to their counterpart without Lipimax (Fig. 6b′). A similar pattern was noted throughout the entire culture period.

### Effect of temperature on cell growth

**Fig. 7:**
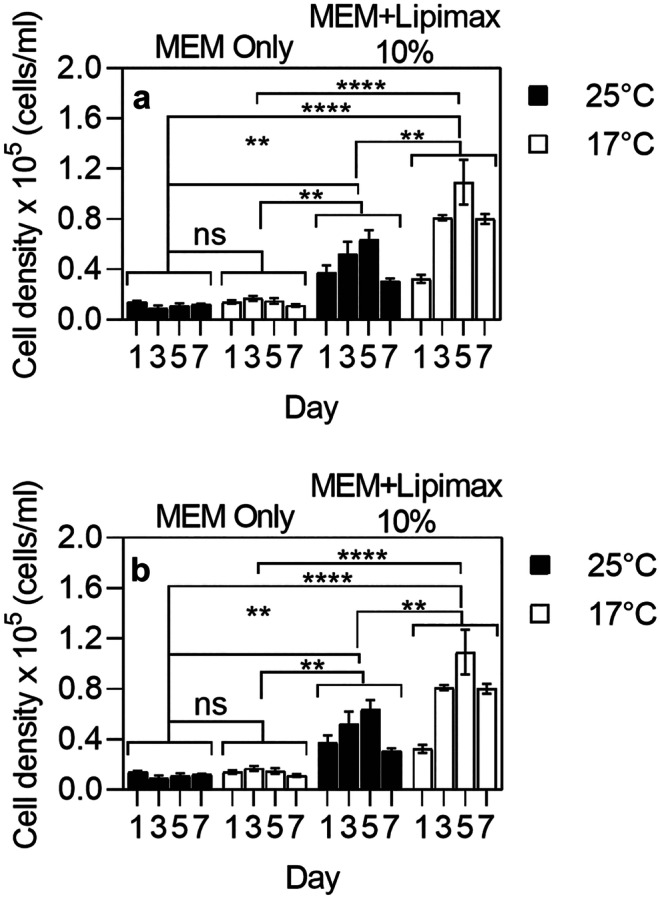
Rhogocyte cells **a** yield and **b** viability in MEM media with and without 10% Lipimax at different temperatures (25 °C and 17 °C). ****P < 0.0001, ***P < 0.005, **P < 0.05, *P < 0.5, ns, not significant

Culture consisting of rhogocyte cells were incubated at 17 °C (refrigerated BOD incubator, Shel Lab) and 25 °C (HERACell 150i CO_2_ incubator, Thermo Scientific) to examine the impact of temperature on their growth. As depicted in Fig. 7, increased cell growth was observed when cultured with 10% Lipimax at 17 °C compared to 25 °C (*P* < 0.005). The maximum growth was achieved by days 5; 7.8 and 4.5-fold at 17 °C and 25 °C, respectively. The result also suggested that the effect of temperature on cell growth was negligible unless Lipimax was added to the media. In addition, the temperature had a minimal effect on cell viability in both treatments. Therefore, optimal cell yield was achieved when the cells were cultured in MEM, supplemented with Lipimax at 17 °C.

### Effect of growth supplements on hemocyanin biosynthesis

**Fig. 8:**
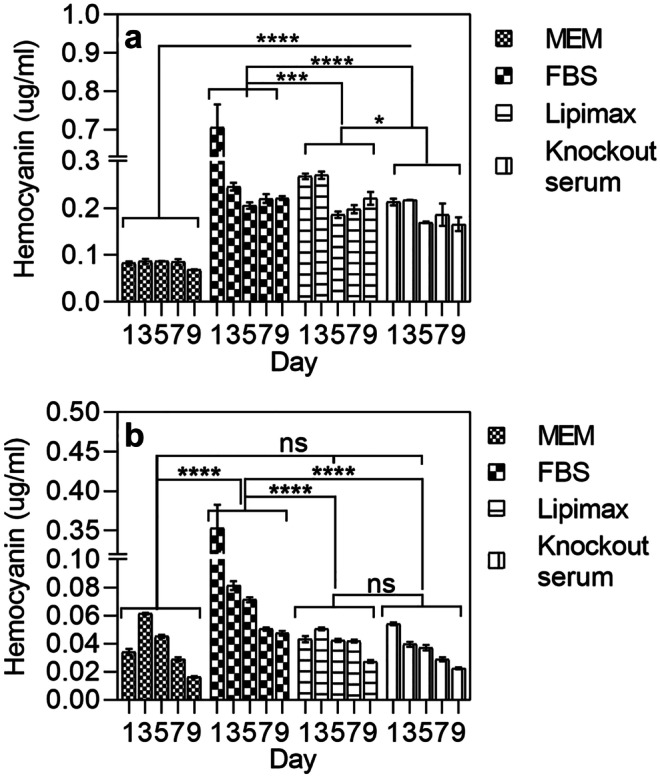
Comparison of hemocyanin content evaluated in media (**a**) and cell pellet (**b**) when cultured with different growth supplements in MEM. ****P < 0.0001, ***P < 0.005, **P < 0.05, *P < 0.5, ns, not significant

Hemocyanin yield in MEM media and cell pellet was investigated using ELISA (Fig. 8). In this study, hemocyanin concentration was measured in the media and cell pellet with or without 10% of growth supplements. Hemocyanin yield increased significantly by at least twofold throughout the culture period in the presence of growth supplements in MEM (Fig. 8a). The highest yield (0.7 µg/ml) in media was recorded on the first day for FBS-supplemented culture, followed by culture with Lipimax and Knockout serum. Each treatment demonstrated a similar trend; a high hemocyanin yield at an early stage (first 24 h) and decrease after 24 h. The protein yield was lowest at day 5 of incubation. At the end of the incubation period, the hemocyanin concentration in the media was reduced to 0.2 µg/ml except for cells cultured in MEM only (below 0.1 µg/ml). On the other hand, hemocyanin concentration obtained from the extracted cell pellet was significantly lower than hemocyanin in the media (Fig. 8b). The amount of protein also decreased gradually over the incubation time. Thus, addition of growth supplements in culture media, resulted in immediate increase of the hemocyanin biosynthesis and release by the rhogocyte cell. However, further increase of hemocyanin biosynthesis in media or cells was not observed.

**Fig. 9:**
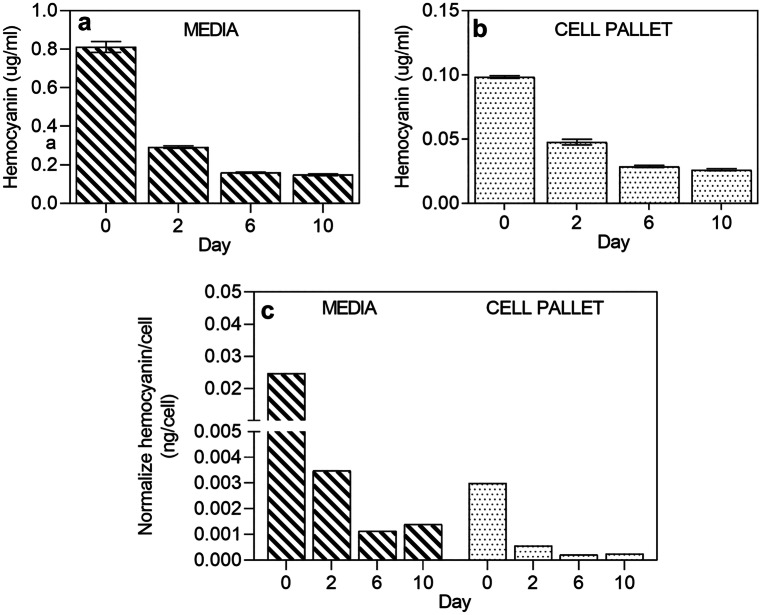
Analyses of hemocyanin biosynthesis and cell yield from days 0 to 10 in MEM media with 10% Lipimax (analysis started 1 h after culture): **a** hemocyanin concentration in the media, **b** hemocyanin concentration in cell pellet, **c** normalised ratio of hemocyanin concentration per cell unit

To correlate the cell growth and hemocyanin yield, another set of study was conducted by using 10% Lipimax (v/v) as the primary growth supplement. Lipimax was chosen because of its optimal growth effect to the cells. The culture media and cells were harvested after 1 h (day 0), days 2, 6 and 10, followed by flow cytometry analysis and ELISA assay. As depicted in Fig. 9, the hemocyanin yield was inversely correlated with cell growth. The hemocyanin concentration approached a maximum in both medium (0.8 µg/ml) and cell pellet (0.1 µg/ml) just an hour after culture (Fig. 9a, b). Hemocyanin concentration, however, decreased rapidly after the first 24 h and reached a stagnant level at 0.2 µg/ml and ~ 0.025 µg/ml after 48 h in media and cell pellets, respectively. Normalised hemocyanin concentration per cell (Fig. 9c) also demonstrated a similar downward trend except for day 10, where the ratio of hemocyanin/cell increased by 23% and 20% in media and cell pellets compared to day 6, respectively.

## Discussion

To the best of our knowledge, the current study is the first attempt to investigate the growth of rhogocyte cells of abalone and their ability to synthesise hemocyanin in vitro. Previous attempt of hemocyanin biosynthesis was demonstrated by Avissar et al. (1981) and Alliel et al. (1983) on arthropod using cell-free synthesis approach. The current study, however, demonstrated the possibility of understanding the rhogocyte cell behaviour and its capability to synthesise hemocyanin in vitro*.* Using a flow cytometer and immunocytochemistry approach, the rhogocyte cells were detected in a heterogeneous primary culture of dissociated abalone mantle tissue. The tissue was selected because it harbours a high population of rhogocyte cells that specialise in producing type 1 hemocyanin (Sairi et al. 2015). The cells are highly abundant in the connective tissue such as under the skin, that made it possible to recover viable cells after dissociation with dispase enzyme. In our study, the effect of different culture conditions on cell growth was studied on a single cell level (rhogocyte cells) using a flow cytometer. Despite the heterogeneous culture, rhogocyte cells were individually detected by anti-hemocyanin antibody accumulated in the ELS. The results of this study suggest that the addition of growth supplements had a significant impact on rhogocyte cell growth, and the effect of this factor is paramount compared to basal medium effect.

In the current study, better cell yield was achieved when cells were cultured in MEM and DMEM media supplemented with growth supplements. In both media, the cells were embedded in extracellular matrix instead of adhering cleanly to the flask polystyrene surface. On the contrary, cells cultured in Leibovitz’s L-15 demonstrated a lower cell growth. The current finding is in contrast to previous studies which suggested L-15 as a suitable media for molluscan cell culture of various tissues (Van Der Merwe et al. 2010; Dessai 2012; Daugavet and Blinova 2015; Suja et al. 2017; Barrick et al. 2018). An earlier study by Suja et al. (2007) on *Haliotis varia* mantle tissue demonstrated that L-15 promotes cell proliferation while M199 enhances cell adherence. It is also worth mentioning that adherent cells without proliferating ability were observed when supplement was omitted from the media.

A comparison between MEM, DMEM, L-15 and F-12 media composition showed that MEM and DMEM include vitamin B_6_ in its aldehyde form (pyridoxal), while in L-15 and F-12, the vitamin in its alcohol form (pyridoxine). Vitamin B_6_ is involved in various metabolic functions such as fatty acid metabolism, immune function, glyconeogenesis, folate metabolism, coenzyme Q synthesis and heme synthesis (Lichtstein et al. 1945; Rall and Meydani 1993; Di Salvo et al. 2011; Huang et al. 2011). Vitamin B_6_ also takes part in the biosynthesis of amino sugars, including the incorporation of amino sugars into protein-linked sugar chains or glycoprotein. Thus, we speculate that rhogocyte cells utilise the vitamin in its aldehyde form more efficiently than its alcohol form. However, further investigation is required to affirm the possibility.

Following media variations, addition of growth supplements significantly affected the cell behaviour in culture. Addition of supplements in the media caused cells to grow in semi-suspended mode due to the presence of the extracellular matrix surrounding the cells. The cells also maintained their rigid structure while trapped in the jelly-like extracellular matrix. As mentioned previously, omitting the serum caused cells to adhere to surfaces but limited their growth. Contrary to available animal cell lines, growth supplements for invertebrate cell culture have yet to be explored extensively. Despite the lack of studies on invertebrate cell culture for decades, the current study demonstrated the positive impact of growth supplements such as Lipimax (a lipoprotein base supplement), fetal bovine serum and Knockout(™) serum towards rhogocyte cells. Furthermore, Lipimax was superior to both FBS and Knockout serum. Lipimax is a highly purified lipoprotein suspension derived from adult bovine serum. It comprised naturally occurring cholesterol, essential fatty acids and phospholipids. The effectiveness of lipid supplementation was previously demonstrated on oyster’s heart cell (Cecil 1969; Domart-Coulon et al. 1994; Cornet 1995). On the other hand, fetal bovine serum were shown to enhance mollusc cell culture (Dessai 2012; Suja et al. 2017; Barrick et al. 2018). High concentration of FBS, however, may cause a detrimental effect to some cells (Kusumoto et al. 1997), whilst, batch to batch variations may also have an impact on cell growth. Knockout serum, on the other hand, is a defined serum-free formulation used in stem cell culture. Garcia-Gonzalo and Belmonte (2008) had successfully stimulated human embryonic stem cell renewal using Knockout serum.

The current study also demonstrated the significant impact of temperature on cell growth where reducing the temperature from 25 to 17 °C significantly increased the growth of rhogocyte cells. Despite studies of optimal culture temperature between 20 and 28 °C for mollusc cell culture (Kusumoto et al. 1997; Suja and Dharmaraj 2005; Dessai 2012; Jayasankar et al. 2018), temperature may vary according to species as suggested by Gilroy and Edwards (1998). According to Gilroy and Edwards’ (1998) study on abalone aquaculture, the preferred temperature for blacklip (*H.* rubra) and greenlip abalone (*H. laevigata*) were between 17 and 18 °C. As temperature increased, the animals began to lose their foothold (equivalent to death) when the temperature reached 25 °C. Despite the positive impact of lower temperature (17 °C) in the current study, growth only occurred when supplement was included in the media. Furthermore, growth supplements also triggered synthesis and release of hemocyanin into the media.

Addition of FBS, Lipimax and Knockout serum also increased the hemocyanin content in the media significantly whilst its concentration remained unchanged in the cells. With supplements, the ratio of hemocyanin in the media was at least 4–7 times higher than the cell pellet. Thus, the supplement contains a key compound that triggers the cells to either release or synthesise more hemocyanin in the media. In the absence of supplement in the basal media, the cells only produce a small amount of hemocyanin. When supplement was included, the cells were triggered to synthesise hemocyanin and release it into the media. Previously, Kokkinopoulou et al. (2015) proposed the colloidosmotic pressure mechanism to explain hemocyanin biosynthesis by rhogocyte cell where accumulation of hemocyanin in ELS caused water to flow into the ELS and built-up pressure. The pressure then induced contraction of the actin-rich peripheries of the cytoplasmic bars to release the ELS content. This study implies that hemocyanin released from ELS was only triggered when growth supplements were included in the media. Our study also illuminated the rapid synthesis of hemocyanin during cell culture depicted in Fig. 8, where the highest concentration of hemocyanin was within the first hour of culturing the rhogocyte cells. The rapid increase of hemocyanin in the culture media after an hour of culture demonstrated the capability of rhogocyte to perform a burst synthesis of hemocyanin when triggered by the growth supplement. If the hemocyanin was stored in the cell at the initial time of culture, the spike of hemocyanin signal should be observable in cell pellet when cultured with basal media only. Thus, it is possible that the growth supplement may contain factors that stimulate the hemocyanin biosynthesis prior to relocation to ELS to undergo the colloidosmotic pressure release. Thus, we inferred that cholesterol, essential fatty acids and phospholipids may play an important role during the culture and biosynthesis process. Rapid biosynthesis of hemocyanin may also be due to the presence of mRNA in the cytoplasm of lower signal population reported in our previous study (Sairi et al. 2015). The exact component involved in triggering the synthesis/release of hemocyanin, however, is still obscure.

The presence of two different populations of cells with different hemocyanin distributions throughout our previous study suggested that the rhogocyte cell populations may include progenitor rhogocyte cells and their mature counterpart that lacks nucleus, resembling the haemopoietic nature of human blood cells (previously coined as resting and active rhogocyte cell in Sairi et al. 2015, respectively). The progenitor rhogocyte cells are able to divide into another progenitor cells and/or mature cells that specialise in hemocyanin biosynthesis. The proposed idea of having progenitor and mature rhogocyte cells is supported by our results depicted in Fig. 6. It is convenient to make a correlation between the cell population with high hemocyanin signal and the progenitor rhogocyte cells as they continue to proliferate throughout the culture period with a lower percentage of cell death. Although the other population also proliferates up to day 7, their cell viability was lower with more than 60% observed cell death. The high cell death may be attributed to the shorter life span of matured rhogocyte cells, especially after a dramatic burst of hemocyanin biosynthesis. A similar idea was already proposed by Sminia and Knaap (1983), who suggested that gastropods do not have specialised hemopoietic organ, but cell proliferation occurs in connective tissue throughout the body. They also hypothesised that round blood cells known as young cells can differentiate into mature spreading cells (Sminia and Knaap 1983). Furthermore, they demonstrated that only a small percentage of the cells actively synthesised DNA based on in vitro and in vivo labelling, which is in agreement with our previous finding (Sairi et al. 2015). The proposed idea was also in agreement with Streit et al. (2005) where the latter demonstrated that both hemocyanin isoform genes were initially expressed by a small number of mesenchymal cells at trochophore and pre-torsional veliger stages but not at a specific organ (Streit et al. 2005). As the abalone grows, the isoform distribution changed to show a certain level of isoform-specific function during the developmental stages. However, the current strategy of detecting the cells based on hemocyanin trapped in extracellular lacunae space also has its bias, due to the fact that the availability of hemocyanin in the lacunae space changes over time as suggested by Kokkinopoulou et al. (2015). They found that hemocyanin was absent in some rhogocytes and suggested that rhogocyte cells were only able to either synthesise or secrete hemocyanin in the extracellular lacunae space at one particular time. Therefore, further investigations using suitable surface markers are necessary to uncover the different subtypes of rhogocyte cells.

## Conclusions

We conclude that rhogocyte cells are most likely proliferated for the first 6 days of culture when grown in media supplemented with 10% Lipimax at 17 °C. Furthermore, the addition of Lipimax in the culture stimulates the cells to synthesise and secrete hemocyanin into the media after an hour of culture. This study demonstrated an inverse relationship between rhogocyte cell growth and hemocyanin concentration in the media. Depletion of the protein during cell growth suggests the possibility of cells recycling the protein in order to continue proliferating. As the protein reached a plateau, the number of cells decreased suddenly and revealed the presence of two types of cell populations, which suggests the existence of progenitor and mature rhogocyte cells. The developed methodology for culturing of abalone rhogocyte cells will facilitate further studies in the biology of rhogocytes, the biosynthesis and complex functionality of hemocyanin.

## Supplementary Information

Below is the link to the electronic supplementary material.

Supplementary file1 (TIF 19448 KB)

## Abbreviations

FITC: Fluorescence isothiocyanate
DAPI: 4′,6-Diamidino-2-phenylindole, dihydrochloride
