# Supplementary figures and images for: Lipoprotein-induced cell growth and hemocyanin biosynthesis in rhogocytes
Source: Cell Tissue Res. 2022 Jan 28;388(2):359–71. doi: 10.1007/s00441-022-03577-1 (PMC9035422; doi:10.1007/s00441-022-03577-1)

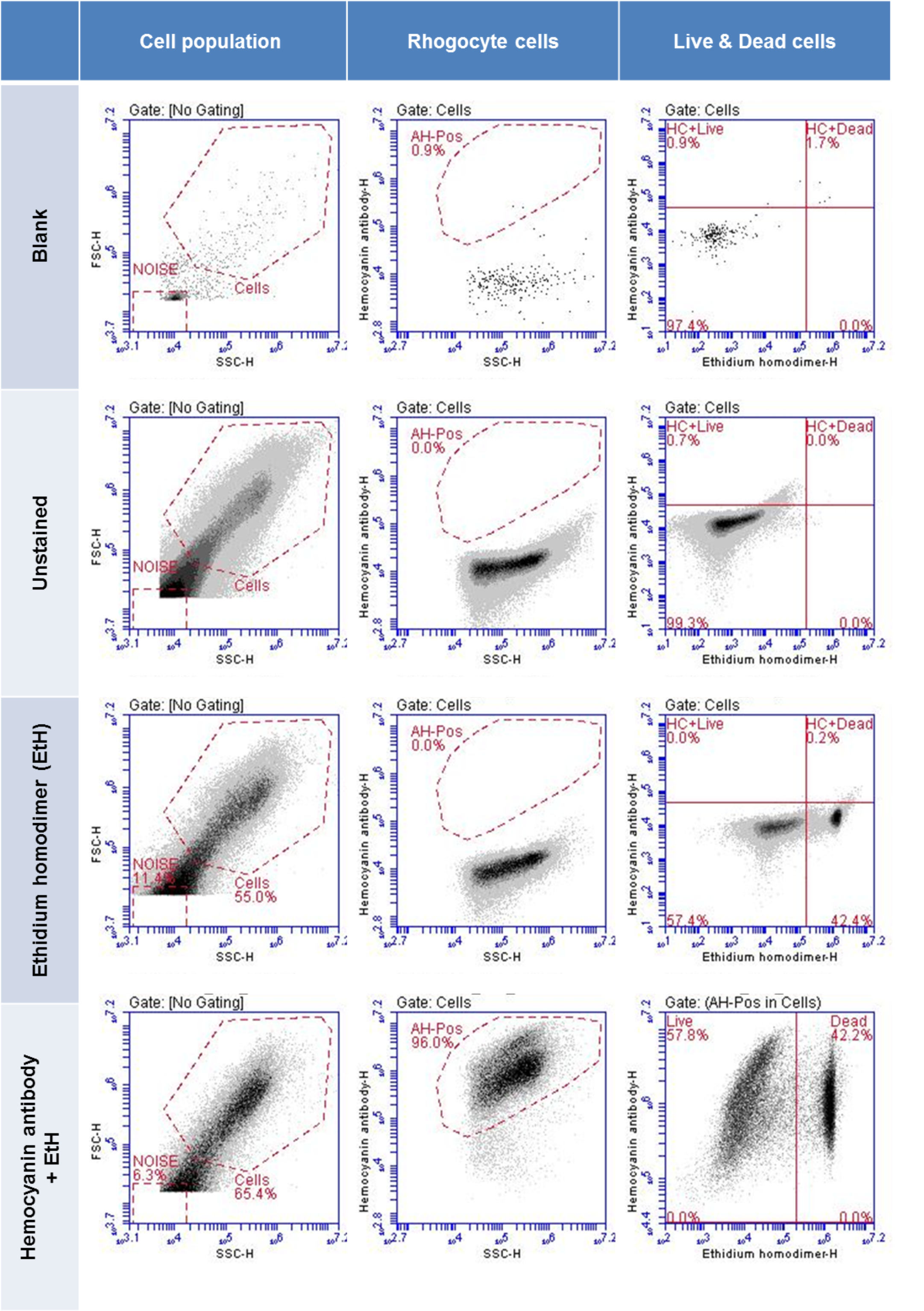

Supplement: Supplementary file 1 — Supplementary file1 (TIF 19448 KB) [file 441_2022_3577_MOESM1_ESM.tif]
